# Postoperative analgesic efficacy of intertransverse process block vs. erector spinae plane block in total laparoscopic hysterectomy: a randomized double-blind trial

**DOI:** 10.3389/fmed.2026.1783174

**Published:** 2026-06-05

**Authors:** Yang Zeng, Qian Chen, Huimin Peng, Di Wu, Shangshi Ji, Huan Li, Feiyun Lu, Haiyun Du, Bin Qian

**Affiliations:** 1 Jiangsu Province Key Laboratory of Anesthesiology and Brain Science, Xuzhou Medical University, Xuzhou, Jiangsu, China; 2 Jiangsu Province Key Laboratory of Anesthesiology, Xuzhou Medical University, Xuzhou, Jiangsu, China; 3 Yancheng Clinical College, Xuzhou Medical University, Yancheng, Jiangsu, China; 4 Department of Anesthesiology, Yancheng First People's Hospital, Yancheng, Jiangsu, China

**Keywords:** erector spinae plane block, intertransverse process block, perioperative analgesia, total laparoscopic hysterectomy, ultrasonic guidance

## Abstract

**Background:**

Due to multiple factors, patients will experience significant postoperative pain following laparoscopic total hysterectomy (TLH), predominantly manifesting as visceral pain. For the first time, we compared the analgesic effects of intertransverse process block (ITPB) and erector spinae plane block (ESPB) in patients undergoing TLH. We hypothesized that ITPB would further reduce the perioperative opioid dosage and improve visceral pain in TLH patients when compared with ESPB.

**Methods:**

This research included 66 patients (18–65 years-of-age) undergoing TLH under general anesthesia. Patients were assigned by randomization to either an ITPB or ESPB group. Ultrasound-guided bilateral intertransverse process blocks at T10-11 were performed for patients in the ITPB group, with 0.375% (20 mL) ropivacaine per side. For the ESPB group, we performed bilateral ultrasound-guided ESPB at the T10 level with 0.375% ropivacaine (20 mL) per side. All patients received total intravenous anesthesia guided by the bi-spectral index (BIS). The primary outcome included total opioid use, expressed as morphine equivalents, within the first 24 h following surgery.

**Results:**

Patients in the ITPB group required significantly lower intraoperative remifentanil doses (mean difference: −328.0 μg, 95% CI: −395.2 to −260.9, *p* < 0.001) and exhibited reduced cumulative opioid consumption within 24 h postoperatively (mean difference: −2.5 mg morphine equivalents, 95% CI: −3.85 to −1.15, *p* < 0.001) when compared to the ESPB group. The ITPB group also exhibited significantly lower Numerical Rating Scale (NRS) scores for visceral pain upon awakening and at 2, 4, 6, 8, and 24 h after surgery when compared to the ESPB group (*p* < 0.001). Similarly, NRS scores for incisional pain were significantly lower in the ITPB group immediately after awakening and at 4, 6, and 8 h postoperatively (*p* < 0.05).

**Conclusion:**

Compared with ESPB, ITPB more effectively reduced perioperative opioid consumption and provided superior and sustained postoperative visceral analgesia for patients undergoing total laparoscopic hysterectomy. In addition, ITPB was associated with improved short-term postoperative recovery quality. In future, studies need to elucidate the underlying mechanisms of ITPB and optimize the volume and concentration of local anesthesia for administration.

## 1 Introduction

Total laparoscopic hysterectomy (TLH) is a minimally invasive surgical technique that is commonly used in the gynecology clinic (1, 2). Compared with traditional form of laparotomy, abdominal hysterectomy, TLH is associated with lower levels of postoperative pain, reduced morbidity, shorter hospital stays, and a more rapid recovery (1). However, patients still experience considerable postoperative pain due to a variety of factors, including preoperative anxiety (3), abdominal wall incisions, pneumoperitoneum, surgical manipulation, and postoperative drain stimulation, with visceral pain representing the predominant component (4). At present, postoperative analgesia predominantly relies upon opioid-based regimens (5). Nevertheless, the administration of opioids can result in significant adverse effects, including urinary retention, respiratory depression (RD), sedation, ileus, and postoperative nausea and vomiting (PONV), which can all delay recovery, thus causing the patient to fail to regain consciousness and delaying discharge from hospital. The development of enhanced recovery after surgery (ERAS) protocols, and advances in ultrasound technology, over the last few years, has led to the wide recognition of ultrasound-guided nerve blocks as a key element of multimodal pain control (6). These techniques can reduce the perioperative consumption of opioids and thereby reduce the incidence of side effects associated with the use of opioids. Furthermore, by interrupting afferent neural transmission, these blocks can modulate the stress response, attenuate activation of the endocrine system, reduce immunosuppression, and promote early postoperative recovery.

Based on anatomical principles, local anesthetics can reduce postoperative somatic and visceral pain when delivered close to the dorsal root ganglia (7). Previously, the preferred option was paravertebral block (PVB), a procedure in which local anesthetics are administered into the paravertebral space surrounding the spinal nerve (7). However, although PVB can create long-term blockade and reliable analgesic effects, this strategy is technically difficult and is associated with the potential for severe complications (8), thus highlighting the need to identify appropriate alternatives. Erector spinae plane block (ESPB) and intertransverse process block (ITPB) have both emerged as potential alternatives for PVB (9, 10). ITPB has recently emerged as a modified form of paravertebral block for postoperative analgesia, with the local anesthetic inserted between the pleura posterior border and the transverse process (TP) (9, 10). Previous studies have demonstrated that ITPB provides non-inferior analgesia when compared with PVB for pediatric cardiac surgery and thoracoscopic procedures (11, 12); there were no significant differences between the two techniques in terms of perioperative opioid use, time to ICU discharge, time to extubation, or Numerical Rating Scale (NRS) scores. However, ITPB was proven to be faster and safer (11, 12). For ESPB, local anesthetics are injected into the erector spinae plane, a fascial layer situated between the erector spinae muscle (ESM) and the underlying TPs. ESPB is associated with reduced opioid usage after lumbar spine surgery (13) and TLH (14). These two block protocols are applied farther away from the pleura and blood vessels than PVB and are therefore, less invasive (9).

The specific analgesic mechanisms provided by ESPB and ITPB have yet to be fully elucidated. Furthermore, previous studies have failed to compare bilateral ITPB with ESPB with regards to analgesia in abdominal visceral surgery. On this basis, we conducted a randomized controlled trial (RCT) to compare the analgesic efficacy of ESPB and ITPB for patients receiving TLH. We hypothesized that ITPB would reduce perioperative opioids and improve visceral pain when compared to ESPB. Our primary outcome was cumulative opioid consumption within 24 h post-surgery, expressed as morphine equivalents (including sufentanil in the PACU and tramadol on the ward).

## 2 Methods

### 2.1 Study design

This prospective RCT enrolled 70 female patients between November 2024 and February 2025. Our research was authorized by the Ethics Committee of Yancheng First People’s Hospital (approval number: 2024-K-356,14/11/2024) and was registered in the Chinese Clinical Trial Registry (ChiCTR2500096111; registration date: 17/01/2025). Our research protocol adhered to the Declaration of Helsinki and all patients provided written and informed consent. The research design and reporting followed the CONSORT guidelines.

The inclusion criteria were as follows: patients scheduled for elective TLH (only patients with benign uterine disease were included, and the surgical procedure consisted of total hysterectomy with bilateral adnexectomy); aged 18–65 years; BMI of 19–28 kg/m^2^; and with an ASA physical status of I, II, or III. The exclusion criteria were as follows: refusal to participate in the study; contraindications to deep nerve block, including allergy to local anesthetics; coagulation disorders (platelet count < 70 × 10^9^/mL and/or an international normalized ratio [INR] > 1.5); injection-site infection; a history of severe hypertension (diastolic blood pressure > 110 mmHg or systolic blood pressure > 180 mmHg); prolonged analgesic use; psychiatric disorders or an inability to complete scoring scales; and clinical inability to perform nerve block due to anatomical difficulty during ultrasound scanning. The withdrawal criteria were as follows: voluntary withdrawal by the patient; intraoperative conversion to another surgical procedure; and loss to follow-up after surgery.

SAS version 9.3 software was used to randomly allocate participants to the ITPB or ESPB group with a 1:1 ratio. The randomization sequence was enclosed in sequentially numbered opaque envelopes, and a designated study coordinator was responsible for storing and distributing the randomization results. The coordinator opened the envelopes sequentially according to allocation, assigned the patients into their respective groups, and prepared the block medications. All nerve blocks were carried out by an anesthesiologist who had over 10 years of working experience; this anesthesiologist did not play a role in the subsequent management of anesthesia. The in-room anesthesiologist responsible for induction and intraoperative management was blinded to group allocation. PACU nurses and anesthesiologists were also blinded. A researcher who had not performed the nerve block procedures or intraoperative management was assigned to conduct postoperative follow-up.

### 2.2 Regional anesthesia technique

Patients were brought to the preparation room 30 min prior to surgery to establish peripheral venous access. We also recorded electrocardiogram (ECG), pulse (P), pulse oxygen saturation (SpO_2_), and bi-spectral index (BIS). Prior to the induction local anesthesia, we established invasive arterial blood pressure monitoring via catheterization, of the radial artery, and oxygen was supplied through a face mask at a flow rate of 3 L/min. Nerve blocks were then administered by an experienced anesthesiologist with patients in the lateral decubitus position. Prior to initiating the preoperative nerve block, midazolam (2 mg) and remifentanil (20 μg) were administered for sedation and analgesia. Povidone-iodine was used to prepare the skin.

**Figure 1:**
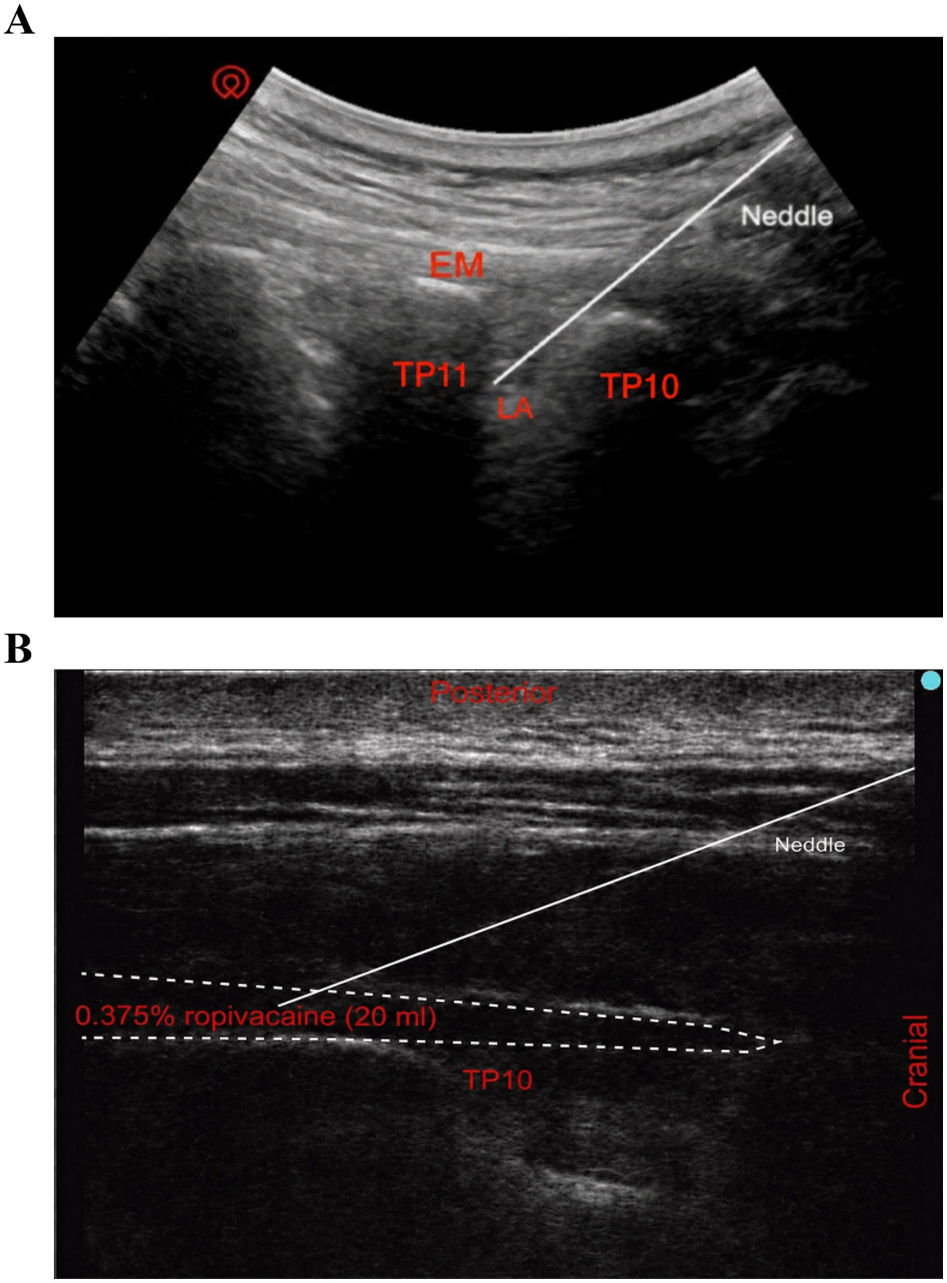
Ultrasound images of two groups. **(A)** Ultrasound image of the intertransverse process block: T10/11 transverse process; **(B)** An ultrasound image of LA was shown in the erector spinae plane. EM, erector spinae muscle; TP, transverse process; LA, local anesthetic; TP, transverse process.

Bilateral T_10-11_ ITPB or bilateral T_10_ ESPB was then performed as follows (Figure 1). In the ITPB group, patients were positioned in the right lateral decubitus. Following routine disinfection and draping, we positioned a low-frequency convex ultrasound probe, covered in a sterile sheath. The probe was inserted into the T_10-11_ intercostal space and then moved medially toward the midline. The needle was advanced from the cranial to the caudal direction toward the intertransverse space via an in-plane approach. Once the needle tip reached the posterior aspect of the superior costotransverse ligament (SCTL) without perforation, the precise position of the tip was proven by visualizing the spread of normal saline (1.0–2.0 mL) within the compartment between the posterior aspect of the SCTL and the anterior surface of the erector spinae fascial plane (9, 10). After confirming the correct needle position, 0.375% ropivacaine (20 mL) was administered at the T_10-11_ level. The same protocol was employed on the contralateral side.

In the ESPB group, patients were positioned in the right lateral decubitus position. Following routine disinfection and draping, a low-frequency convex ultrasound probe, covered with a sterile sheath, was positioned in a longitudinal sagittal orientation approximately 3 cm lateral to the T_10_ spinous process. After identifying the trapezius muscle, rhomboid major muscle, ESM, and the TP, a peripheral nerve block needle was advanced from a cranial to caudal position. Upon contact of the needle with the posterior surface of the TP, we confirmed the absence of blood by aspiration, followed by the injection of normal saline (0.5–1 mL) to perform a hydro-dissection test. After confirming the correct needle position, 0.375% ropivacaine (20 mL) was injected, and adequate spread of the injectate was verified under ultrasound. A similar protocol was conducted on the contralateral side (total: 40 mL).

### 2.3 General anesthesia technique

Following completion of the nerve block, approximately 10 to 20 min later, the patient was transferred to the operating room and underwent standardized monitoring prior to general anesthesia. Upon entering the operating room, general anesthesia was induced intravenously with propofol (2–3 mg/kg), remifentanil (1.5 μg/kg), and rocuronium (0.6 mg/kg), followed by the insertion of a size 3 laryngeal mask airway. Mechanical ventilation was initiated after the laryngeal mask had been placed.

Propofol was continuously infused to maintain anesthesia at 3–8 mg/(kg·h), targeting a BIS of 40–60. Prior to skin incision, we administered 5 μg of sufentanil and 1 μg/kg of remifentanil. Remifentanil infusion was initiated at 0.08 μg/(kg·min), and the pump rates were adjusted intraoperatively within 0–0.2 μg/(kg·min) according to heart rate and blood pressure to maintain changes within ± 20% of the baseline compared with pre-induction values, up to 0.2 μg/(kg·min). When blood pressure exceeded 20% above baseline, we injected urapidil (5 mg) intravenously. If blood pressure fell 20% below baseline and the heart rate was > 40 beats/min, we injected 8 μg of norepinephrine intravenously. When heart rate fell below 40 beats/min, we administered 6 mg of ephedrine intravenously. For severe bradycardia (a heart rate < 40 beats/min), we administered atropine (0.5 mg) intravenously, and for tachycardia (a heart rate >100 beats/min), we injected esmolol (0.5 mg/kg) intravenously. Rocuronium was intermittently supplemented intraoperatively to ensure sufficient muscle relaxation. Thirty minutes prior to the completion of surgery, we administered flurbiprofen axetil (50 mg) and palonosetron (0.25 mg).

### 2.4 Postoperative management and assessment

Following the completion of surgery, all patients were administered with intravenous sugammadex (2–4 mg/kg). Following the recovery of spontaneous breathing, the laryngeal mask airway was withdrawn. Following transfer to the PACU, NRS scores were evaluated every 10 min, and sufentanil (2.5 μg) was administered when NRS was ≥ 3. If the NRS score remained ≥ 3, an additional dose of sufentanil (2.5 μg) was administered each time until the NRS fell below a grade of 3. The Steward score was assessed by an experienced anesthesiologist, and a Steward score > 4 indicated readiness for discharge from the PACU (15).

Two hours after returning to the ward, we inserted a diclofenac sodium suppository (50 mg). Beginning on the following day, we commenced oral acetaminophen (0.5 g three times daily). When the NRS was > 3, we administered intravenous tramadol (50 mg) for rescue analgesia. The maximum daily dose of tramadol was 400 mg.

### 2.5 Outcomes

The primary outcome was morphine equivalent opioid consumption within 24 h of surgery (including sufentanil administered in the PACU and rescue tramadol), calculated according to the following conversion formula: 1 mg morphine (iv) = 1 μg sufentanil (iv) = 10 mg tramadol (iv) (16).

The secondary outcomes were intraoperative remifentanil consumption; NRS scores for resting abdominal wall incision pain and visceral pain at key postoperative time points: T0 (immediate full awakening), T1 (2 h), T2 (4 h), T3 (6 h), T4 (8 h), and T5 (24 h); the administration frequency for rescue opioids (tramadol); the occurrence of intraoperative heart rate/mean arterial pressure (HR/MAP) fluctuations exceeding ±20% from baseline; overall postoperative quality of recovery at 24 h according to the QoR-15 (17, 18); postoperative complication rates (vomiting, nausea, pruritus, dizziness, and other adverse reactions); complications associated with the nerve block, including local anesthetic systemic toxicity and nerve injury; postoperative time to first ambulation; and the duration of hospitalization.

### 2.6 Sample size and data analysis

According to preliminary data from 20 patients (*n* = 10/group), the morphine equivalent opioid consumption within 24 h post-surgery was 3 ± 2.18 mg for the ITPB group and 4.5 ± 1.87 mg for the ESPB group. Sample size calculation was implemented with G*Power v3.1.9 software, assuming a power of 80% and an *α* level of 0.05, thus resulting in for need for 27 participants per group. Given a 20% dropout rate, 33 participants were needed per group, yielding a final total of 66 patients.

Statistical tests were performed with SPSS version 25.0. Continuous data following or approximating a normal distribution are presented as mean ± standard deviation, and between-group comparisons were implemented with independent-sample t tests. The Shapiro–Wilk test was used to evaluate the normality of continuous data. Skewed data are presented as median (Q_1_, Q_3_), and generalized estimating equations were used to evaluate overall group, time, and interaction effects. For variables not conforming to a normal distribution, the Mann–Whitney U test was applied for intergroup comparisons, and the Friedman rank test was utilized for repeated measurements over time. Multiple comparisons were adjusted using the Bonferroni method, with statistical significance set at *p* < 0.05.

## 3 Results

### 3.1 Sample screening flow

**Figure 2:**
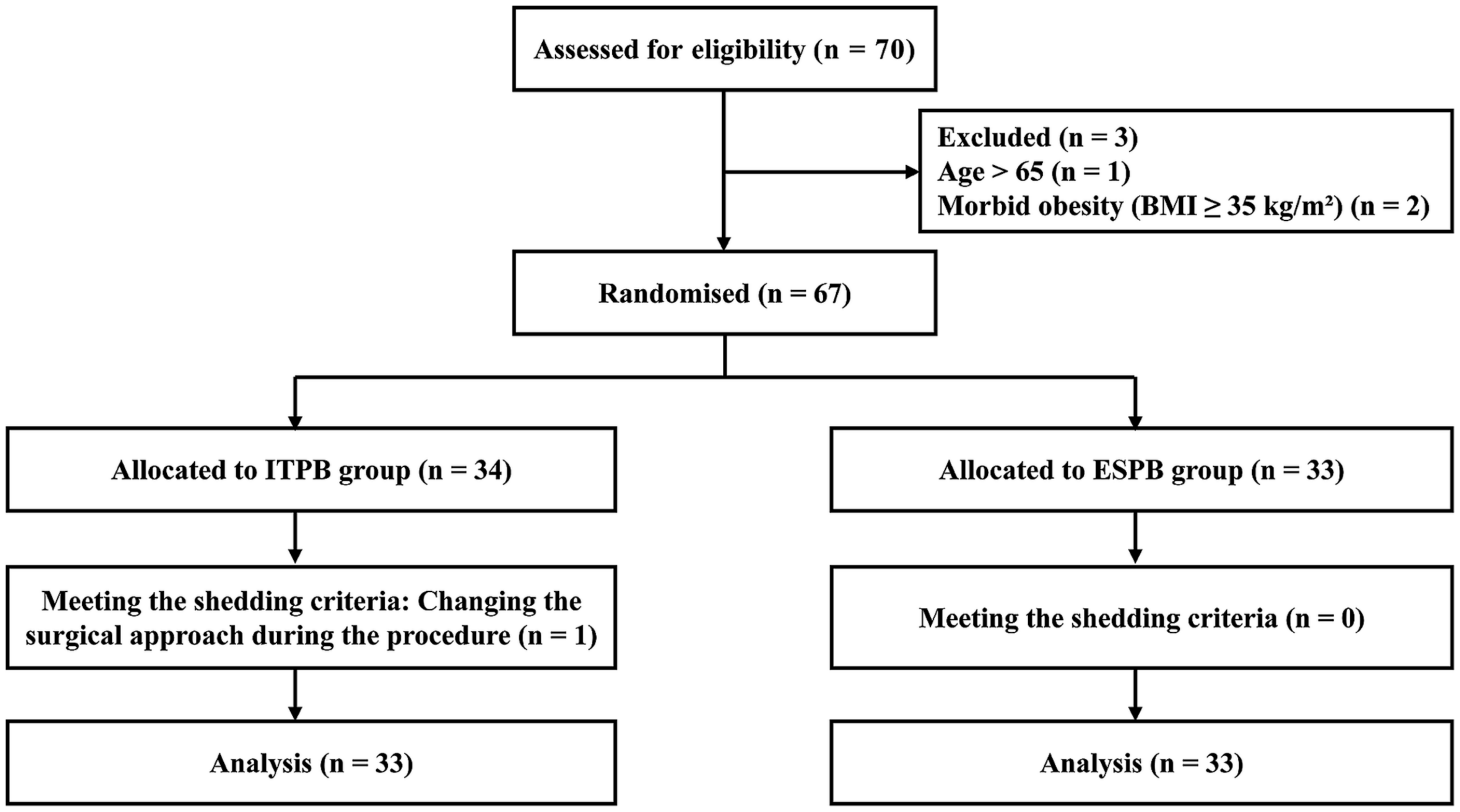
Flow diagram showing the patient screening process. *ITPB*, Intertransverse process block; *ESPB*, erector spinae plane block.

Figure 2 shows a flowchart depicting the sample screening process. Seventy patients were initially evaluated for eligibility. Three individuals were excluded: one due to age (68 years) and two due to morbid obesity (BMI ≥ 35 kg/m^2^). Consequently, 67 patients were enrolled and randomized, with 34 and 33 patients allocated to the ITPB and ESPB groups, respectively. One patient met the withdrawal criteria due to an intraoperative change in surgical method. The remaining 66 patients completed follow-up, with no losses reported.

### 3.2 Comparisons of baseline features between the two groups

No significant differences were detected between the two groups in terms of age, BMI, ASA physical status, comorbidities, or the duration of surgery (Table 1; *p* > 0.05).

**Table 1:** Comparison of general characteristics between the two groups.

Group	Age (years)	BMI (kg/m^2^)	ASA class (II)	Duration of surgery (min)	Hypertension (n)	Diabetes (n)
ESPB group	49.88 ± 4.85	23.50 (23.20, 24.10)	33	70.00 (60.00, 80.00)	6	2
ITPB group	50.18 ± 3.93	23.90 (23.30, 24.50)	33	70.00 (62.00, 85.00)	5	1
$x_{/t\;\text{value}}^{2}$	−0.279	−1.079	0.000	−0.880	0.109	0.000
*p* value	0.781	0.287	1.000	0.379	0.280	1.000

Data are expressed as median and interquartile range [M (P25, P75)] or mean ± standard deviation $\left(\bar{x}\pm s\right)$ . ASA, American Society of Anesthesiologists; ITPB, Intertransverse process block; ESPB, erector spinae plane block.

### 3.3 Comparison of intraoperative and postoperative opioid consumption

**Figure 3:**
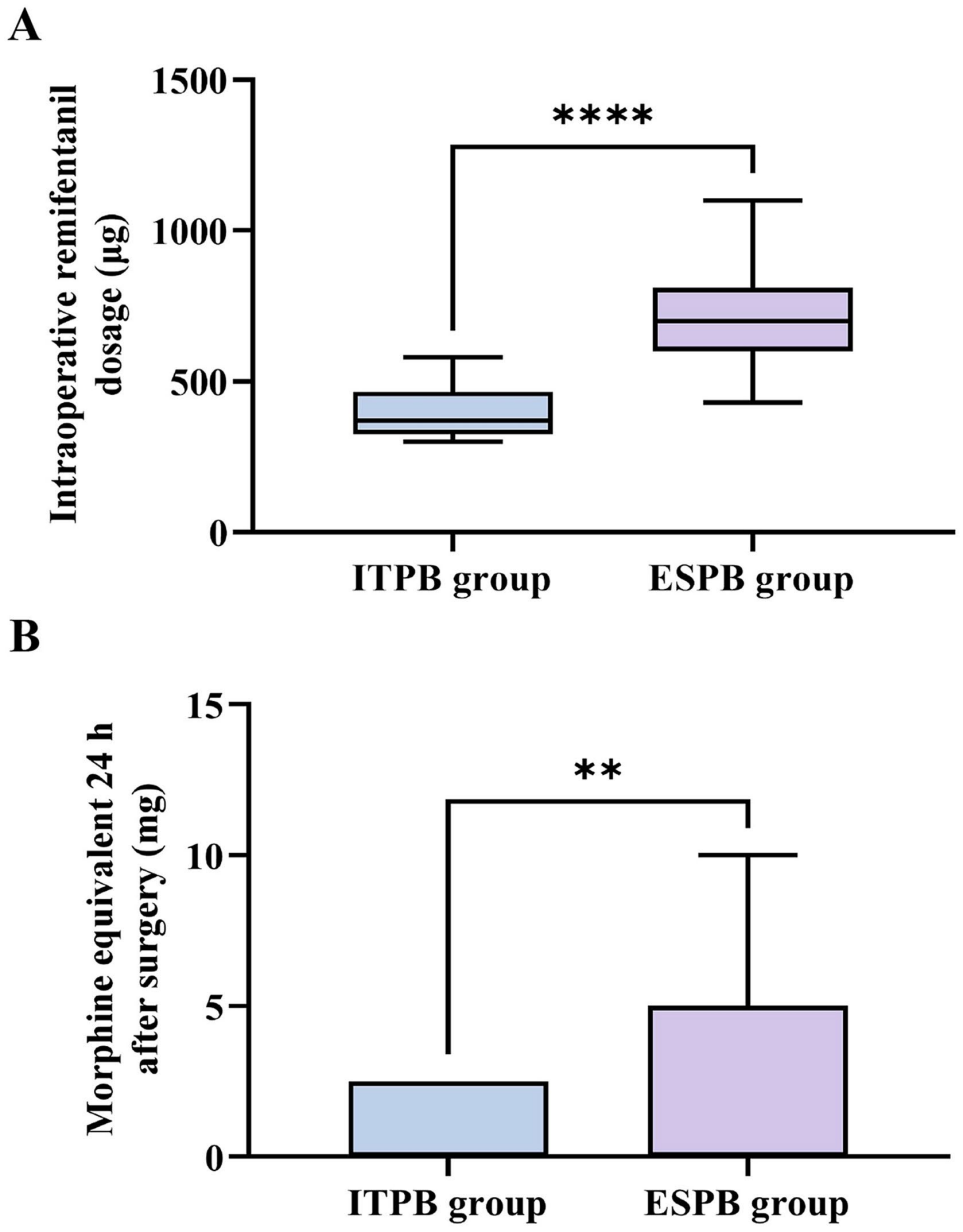
Opioid consumption in the two groups. **(A)** Intraoperative remifentanil consumption in the two groups; **(B)** Morphine equivalent opioid consumption within 24 h in the two groups. *p* < 0.01, *p* < 0.001. *ITP* B, Intertransverse process block; *ESPB*, erector spinae plane block.

The 24-h postoperative morphine equivalent opioid consumption was significantly lower in the ITPB group than in ESPB group (*p* < 0.01). Intraoperative remifentanil consumption was also significantly lower in the ITPB group than in ESPB group (*p* < 0.01). There was no significant difference between the two groups in terms of the number of patients in group that needed for tramadol for rescue analgesia (Table 2; Figure 3).

**Table 2:** Postoperative 24-h opioid consumption in the two groups.

Group	Remifentanil consumption (μg)	Morphine equivalent opioid consumption within 24 h (mg)	Tramadol (n)
ESPB group	700.00 (600.00, 800.00)	5.00 (2.50, 5.00)	3
ITPB group	370.00 (330.00, 450.00)	0.00 (0.00, 2.50)	3
$x_{/t\;\text{value}}^{2}$	−6.507	−3.126	
*p* value	< 0.001	< 0.001	1

Data are expressed as median and interquartile range [M (P25, P75)]. ITPB, Intertransverse process block; ESPB, erector spinae plane block.

### 3.4 Comparison of postoperative NRS scores

**Figure 4:**
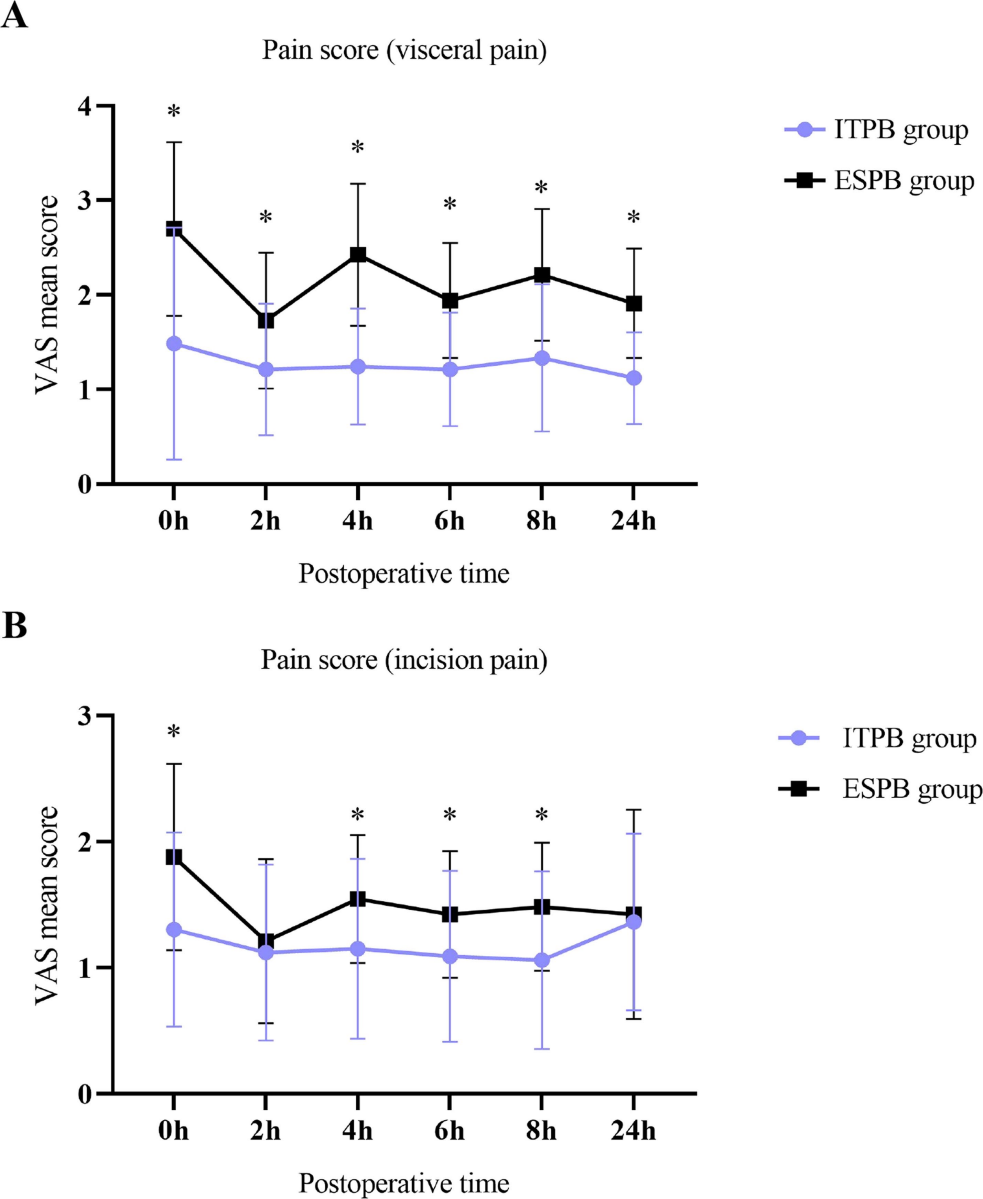
Comparison of pain NRS scores. **(A)** Comparison of visceral pain NRS scores between ITPB and ESPB groups; **(B)** Comparison of incision pain NRS scores between ITPB and ESPB groups. *p < 0.05. *ITPB*, Intertransverse process block; *ESPB*, erector spinae plane block; *NRS*, numerical rating scale.

Next, we compared NRS pain scores for visceral pain and incision pain between ITPB and ESPB. At full awakening and at 2, 4, 6, 8, and 24 h post-surgery, the visceral pain NRS scores were significantly lower in the ITPB group than in the ESPB group (*p* < 0.001; Table 3; Figure 4A). Similarly, the NRS scores for incision pain at full awakening, 4, 6, and 8 h post-surgery were significantly lower in the ITPB group than in the ESPB group (*p* < 0.05; Table 4). No significant differences were identified between the two groups with regards to incision pain NRS scores between the two groups at 2 and 24 h (Table 4; Figure 4B).

**Table 3:** NRS scores for visceral pain within 24 h after surgery in the two groups.

Time point	ITPB group (*n* = 33)	ESPB group (*n* = 33)	Z	*p*	Wald χ^2^ (between groups)	*p* value
T_0_	1.00 (1.00, 3.00)	2.00 (2.00, 3.00)^bdf^	−3.778	< 0.001	40.407	< 0.001
T_1_	1.00 (1.00, 2.00)	2.00 (1.00, 2.00)^ace^	−2.991	< 0.001		
T_2_	1.00 (1.00, 2.00)	2.00 (2.00, 3.00)^b^	−5.425	< 0.001		
T_3_	1.00 (1.00, 2.00)	2.00 (2.00, 2.00)^a^	−4.215	< 0.001		
T_4_	1.00 (1.00, 2.00)	2.00 (2.00, 3.00)^b^	−4.174	< 0.001		
T_5_	1.00 (1.00, 2.00)	2.00 (2.00, 2.00)^a^	−5.168	< 0.001		
χ^2^	7.460	54.237				
*p* value	0.189	< 0.001				
Wald χ^2^ (time effect)	45.726					
*p* value	< 0.001					
Wald χ^2^ (interaction)	23.985					
*p* value	< 0.001					

Data are expressed as median and interquartile range [M (P_25_, P_75_)]. a: compared with T_0_, *p* < 0.05; b: compared with T_1_, *p* < 0.05; c: compared with T_2_, *p* < 0.05; d: compared with T_3_, *p* < 0.05; e: compared with T_4_, *p* < 0.05; f: compared with T_5_, *p* < 0.05. Generalized estimating equations were used to evaluate overall group, time, and interaction effects and the Friedman rank test was utilized for repeated measurements across time. All pairwise comparisons were adjusted using the Bonferroni correction. T_0_ (immediate full awakening), T_1_ (2 h after surgery), T_2_ (4 h after surgery), T_3_ (6 h after surgery), T_4_ (8 h after surgery), T_5_ (24 h after surgery). NRS, Numerical Rating Scale; ITPB, Intertransverse process block; ESPB, erector spinae plane block.

**Table 4:** NRS scores for incision pain within 24 h after surgery in the two groups.

Time point	ITPB group (*n* = 33)	ESPB group (*n* = 33)	Z	*p*	Wald χ^2^ (between groups)	*p* value
T_0_	1.00 (1.00, 2.00)	2.00 (1.00, 2.00)^b^	−2.713	0.007	3.803	0.051
T_1_	1.00 (1.00, 2.00)	1.00 (1.00, 2.00)^a^	−0.511	0.609		
T_2_	1.00 (1.00, 2.00)	2.00 (1.00, 2.00)	−2.279	0.023		
T_3_	1.00 (1.00, 2.00)	1.00 (1.00, 2.00)	−2.017	0.044		
T_4_	1.00 (1.00, 2.00)	1.00 (1.00, 2.00)	−2.490	0.013		
T_5_	1.00 (1.00, 2.00)	1.00 (1.00, 2.00)	−0.257	0.797		
χ^2^	24.333	33.864				
*p* value	< 0.001	< 0.001				
Wald χ^2^ (time effect)	16.122					
*p* value	0.003					
Wald χ^2^ (interaction)	12.483					
*p* value	0.014					

Data are expressed as median and interquartile range [M (P_25_, P_75_)]. a: compared with T_0_, *p* < 0.05; b: compared with T_1_, *p* < 0.05. Generalized estimating equations were used to evaluate overall group, time, and interaction effects and the Friedman rank test was utilized for repeated measurements across time. All pairwise comparisons were adjusted using the Bonferroni correction. NRS, Numerical Rating Scale; ITPB, Intertransverse process block; ESPB, erector spinae plane block.

### 3.5 Comparison of other secondary outcomes

No significant differences were identified between the two groups in terms of the occurrence of intraoperative HR/MAP fluctuations exceeding 20% of baseline, the incidence of postoperative complications (e.g., dizziness, vomiting and nausea), block-associated complications (e.g., local anesthetic systemic toxicity and nerve injury, time to first ambulation post-surgery, or the length of hospitalization; Table 5; *p* > 0.05). Both the ESPB and ITPB groups achieved high postoperative QoR-15 scores (mean ± standard deviation: 139.00 ± 2.33 vs. 141.03 ± 2.31). However, similar incidences of QoR-15 scores were detected between the two groups (*p* = 0.809).

**Table 5:** Comparison of other secondary outcome measures between the two groups.

Group	HR/MAP exceeding 20% of baseline (n)	Time to first ambulation (h)	QoR-15 score at 24 h	Postoperative adverse events (n)		Block-related complications (n)	Length of hospital stay (days)
				PONV	Dizziness		
ESPB group	11	9.00 (9.00, 10.00)	139.00 ± 2.33	5	3	0	5.00 (5.00, 6.00)
ITPB group	5	9.00 (8.00, 9.50)	141.03 ± 2.31	2	1	0	5.00 (4.00, 5.00)
$x_{/t\;\text{value}}^{2}$	2.970	−1.815	−3.552	0.287			−1.696
*p* value	0.085	0.070	0.809	0.766			0.090

Data are expressed as median and interquartile range [M (P_25_, P_75_)]. MAP, Mean Arterial Pressure; QoR-15, 15-item quality of recovery scale; ITPB, Intertransverse process block; ESPB, erector spinae plane block.

## 4 Discussion

Compared with traditional open abdominal hysterectomy, TLH can significantly reduce abdominal somatic pain. However, due to the tissue injury caused by intraoperative CO_2_ pneumoperitoneum and surgical manipulation, patients often experience pronounced visceral pain after surgery, thus prolonging postoperative recovery time and reducing patient satisfaction (4).

First described in 2016, ESPB is a fascial plane block that involves the insertion of local anesthetic beneath the ESM, which can extend to the paravertebral space and produce analgesia by blocking the dorsal and ventral spinal nerve rami. ESPB was initially used to treat thoracic neuropathic pain (10), although subsequent studies have reported the application of ESPB for analgesia in both thoracic surgery (19) and breast reconstruction (20). In addition, ESPB has been shown to reduce the amount of opioid administered following lumbar spine surgery (13) and TLH (14). However, the potential pathway by which local anesthetics traverse the deep layer of the ESM to reach the thoracic paravertebral space (TPVS) has yet to be fully elucidated. In a previous study, Forero and co-workers administered methylene blue dye into the deep layer of the ESM in a fresh cadaver under ultrasound guidance; dissection revealed spread of the dye to both the ventral and dorsal rami within the paravertebral space at the targeted levels (10). Conversely, Jason et al. simulated ESPB in 10 fresh cadavers (20 sides) using the surface of the TP as a key landmark. Of the 20 injections, only one produced dye staining in both the ventral and dorsal rami, resulting in a success rate of only 5% (21). It is possible that dense and complex anatomical structures, such as the semispinalis muscle, multifidus muscle, and the rotatores attached around the TP may have impeded the spread of dye toward the paravertebral and intercostal spaces.

ITPB is a modified paravertebral block that was first proposed in 2017 (22). In ITPB, local anesthetics are injected between the pleura and the TP, spreading to the ventral and dorsal rami of the paravertebral space through fenestration within the SCTL at the injection level. ITPB provides effective analgesia for pediatric cardiac surgery (11), thoracoscopic surgery (12), lumbar decompression (13), major breast cancer surgery (23), and gastric cancer surgery (24). Varela et al. injected local anesthetic along with methylene blue and found that ITPB not only stained the ESM but also produced multi-level staining of the spinal nerves (25). In the present study, we selected the T10-11 level to achieve cranio-caudal spread of the injectate to cover the surgical range of TLH and thus provide effective analgesia. The final needle position for ITPB lies between adjacent TPs, penetrating the deep fascia of the ESM but remaining posterior to the SCTL, a region known as the retro-SCTL space. A previous three-dimensional (3D) micro-computed tomography (CT) and histological investigation revealed the complex architecture of the thoracic intertransverse area and verified that the retro-SCTL space, the target location for ITPB, possesses anatomical pathways that potentially communicate with the TPVS (26). Local anesthetics may diffuse through tissue planes to the TPVS and inhibit sympathetic activity, thereby alleviating visceral pain. The more reliable and consistent entry of local anesthetics into the paravertebral space with ITPB compared with ESPB, and the broader blockade of the dorsal root ganglia, may explain why ITPB reduced intraoperative opioid requirements to maintain hemodynamic stability and further reduced perioperative opioid consumption in the present study. This mechanism might also explain the lower visceral pain NRS scores at 24 h postoperatively in the ITPB group relative to the ESPB group.

Both ESPB and ITPB are relatively simple to perform, with needle tips kept safely away from the pleura and without the need to puncture the SCTL, thus allowing for an effective depth of insertion while minimizing puncture-related risks. Xu et al. previously demonstrated that ITPB carries a low risk of injury to local vessels, the pleura, or nerves, thus avoiding potential complications associated with paravertebral or epidural blocks (12). These findings concur with those of our present study as no block-related complication were detected in either of the ESPB and ITPB groups.

Early postoperative recovery is a major focus in perioperative care, and the QoR-15 scale is commonly employed clinically to investigate the early postoperative recovery quality. Fatigue and anxiety in patients approaching surgery are known to influence the accuracy of preoperative QoR-15 scoring (27). In the present study, we failed to evaluate preoperative QoR-15 scores; therefore, no baseline was available for comparison with postoperative scores. However, both the ESPB group and the ITPB group achieved high postoperative QoR-15 scores; this observation may be related to the incorporation of the ERAS concept, multi-modal analgesia, and the perioperative management strategies designed to enhance postoperative recovery.

In the present study, no significant disparity was observed between the groups with regards to postoperative complication rates. This may be because the sample size of this work depended on calculations based on the primary outcome measure. Neither group experienced postoperative nausea or vomiting; this may be attributed to the use of only small doses of sufentanil, the proactive intraoperative administration of antiemetics, and postoperative multi-modal analgesia based primarily on non-steroidal anti-inflammatory drugs. The time to first ambulation following surgery did not differ significantly between the two groups, potentially because surgeons routinely encourage patients to ambulate on the first postoperative day.

Although the 5 mg difference in morphine dosage and insufficient 1-point reduction in NRS scores between the two groups in this study appears small (28–30), even a minimal reduction in opioid and NRS scores use within a multimodal analgesia regimen holds significant importance. This reduction contributes to a cumulative decrease in opioid-related adverse effects and aligns with the principles of ERAS. The reduction in morphine dosage observed in this study supports the goal of minimizing opioid exposure, a core element of ERAS protocols. The ITPB group demonstrated significantly lower visceral pain NRS scores than the ESPB group at all measurement time points (2, 4, 6, 8, and 24 h), along with significantly lower incisional pain scores at multiple time points (2 and 24 h). The persistence of this analgesic advantage, particularly for the predominant visceral pain component, may enhance patient comfort and satisfaction during the critical early recovery phase. The synergistic effect observed in this study indicates a tangible clinical benefit: reduced opioid requirements coexisting with improved pain scores. This synergistic effect may reduce the risk of opioid-induced side effects (such as nausea, intestinal obstruction, etc.) while providing superior pain control, thereby promoting earlier ambulation and recovery. Therefore, these findings support the clinical significance of ITPB as an essential component of multimodal analgesia strategies for laparoscopic hysterectomy.

This research has several limitations that need to be considered. First, nerve blocks were performed under sedation, and we did not check the dermatomal map or start times; thus, both groups may have included patients with incomplete blocks. Second, we did not evaluate block performance time; therefore, we did not acquire data relating to whether ITPB was simpler to perform than ESPB for postoperative analgesia. Third, the dose of local anesthetic used in this study was 40 mL of 0.375% ropivacaine; it remains unknown as to whether this represents the optimal volume or concentration for TLH. Fourth, postoperative recovery was evaluated only during the first 24 h; we did not evaluate long-term recovery. Finally, this study only considered a small study cohort and was based in a single center. Future work should include a longer follow-up period and a larger sample size and aim to identify the precise mechanisms of action and the precise clinical benefits of ITPB.

## 5 Conclusion

Compared with ESPB, ITPB can further reduce the consumption of perioperative opioids and provide stable and effective analgesia for postoperative visceral pain along with good recovery over the short-term. Therefore, we recommend incorporating ITPB into multi-modal perioperative analgesia protocols for TLH. In addition, for visceral surgery, ITPB appears to be a more suitable alternative to PVB than ESPB. Further research is now required to clarify the specific mechanisms of ITPB and to optimize the volume and concentration of local anesthetic administration.

## Data Availability

The original contributions presented in the study are included in the article/supplementary material, further inquiries can be directed to the corresponding authors.
